# Persistence of lung inflammation and lung cytokines with high-resolution CT abnormalities during recovery from SARS

**DOI:** 10.1186/1465-9921-6-42

**Published:** 2005-05-11

**Authors:** Chun-Hua Wang, Chien-Ying Liu, Yung-Liang Wan, Chun-Liang Chou, Kuo-Hsiung Huang, Horng-Chyuan Lin, Shu-Min Lin, Tzou-Yien Lin, Kian Fan Chung, Han-Pin Kuo

**Affiliations:** 1 Department of Thoracic Medicine II, Chang Gung Memorial Hospital, Taipei, Taiwan; 2 Department of Diagnostic Radiology, Chang Gung Memorial Hospital, Taipei, Taiwan; 3 Division of Pediatric Infectious Diseases, Chang Gung Children's Hospital, Taipei, Taiwan; 4 National Heart & Lung Institute, Imperial College & Royal Brompton Hospital, London, UK

**Keywords:** SARS, alveolar macrophages, T lymphocyte, coronavirus, cytokines, bronchoalveolar lavage

## Abstract

**Background:**

During the acute phase of severe acute respiratory syndrome (SARS), mononuclear cells infiltration, alveolar cell desquamation and hyaline membrane formation have been described, together with dysregulation of plasma cytokine levels. Persistent high-resolution computed tomography (HRCT) abnormalities occur in SARS patients up to 40 days after recovery.

**Methods:**

To determine further the time course of recovery of lung inflammation, we investigated the HRCT and inflammatory profiles, and coronavirus persistence in bronchoalveolar lavage fluid (BALF) of 12 patients at recovery at 60 and 90 days.

**Results:**

At 60 days, compared to normal controls, SARS patients had increased cellularity of BALF with increased alveolar macrophages (AM) and CD8 cells. HRCT scores were increased and correlated with T-cell numbers and their subpopulations, and inversely with CD4/CD8 ratio. TNF-α, IL-6, IL-8, RANTES and MCP-1 levels were increased. Viral particles in AM were detected by electron microscopy in 7 of 12 SARS patients with high HRCT score. On day 90, HRCT scores improved significantly in 10 of 12 patients, with normalization of BALF cell counts in 6 of 12 patients with repeat bronchoscopy. Pulse steroid therapy and prolonged fever were two independent factors associated with delayed resolution of pneumonitis, in this non-randomized, retrospective analysis.

**Conclusion:**

Resolution of pneumonitis is delayed in some patients during SARS recovery and may be associated with delayed clearance of coronavirus, Complete resolution may occur by 90 days or later.

## Introduction

Severe acute respiratory syndrome (SARS) has affected more than 8 thousand patients in 22 countries causing 774 deaths between July 2002 and September 2003 [1]. SARS-associated Coronavirus (SARS-CoV) has been identified as the causative agent [2]. Typical clinical manifestations include fever, cough, dyspnea and rapid progression of pulmonary infiltration or consolidation [3]. The mean mortality rate is 9.6% [1], mostly attributed to hypoxemic respiratory failure. In the acute phase, typical pathological findings in the lungs include mononuclear cells infiltration, alveolar cell desquamation and hyaline membrane formation [4]. Those mononuclear cells may develop into multinucleated giant cells [4]. Proinflammatory cytokines released by alveolar macrophages may play a prominent role in the pathogenesis in SARS [5]. Marked elevation of inflammatory cytokines such as IL-1, IL-6 and IL-12, of the Th1 cytokine, IFN-γ, and of chemokines IL-8, monocyte chemoattractant protein-1 (MCP-1), and IFN-α-induced protein-10 (IP-10) have been reported [6]. High resolution Computed tomography (HRCT) findings at presentation include as unilateral or bilateral ground-glass opacities or focal unilateral or bilateral areas of consolidation [7-9]. Such residual abnormalities have been described also after discharge from hospital at 36.5 days and at 6-months [10,11]. However, limited information is available on recovery of inflammatory abnormalities during recovery from SARS, particularly at 60 days and beyond..

In the current study, we conducted a study to examine HRCT changes in patients who recovered from the acute phase of SARS at days 60 and 90, and measured the associated inflammatory profiles directly by examining bronchoalveolar lavage fluid (BALF). We also examined the presence of coronavirus in BALF. We found persistence of HRCT abnormalities and of lung inflammation at day 60, and determined retrospectively the potential influence of pulse corticosteroid therapy in this process.

## Methods

### Study subjects

Twelve (9 women and 3 men, aged 18 to 51 years) of 28 confirmed SARS patients who were treated in Chang Gung Memorial Hospital in Taiwan between April and May 2003 during the last epidemic of SARS in Taiwan, agreed to participate in this study. All the patients met the modified Centers for Disease Control and Prevention (CDC) case definition of SARS [12]. SARS was confirmed by either positive real-time polymerase chain reaction (PCR) assays or elevated serum anti-coronavirus antibody by ELISA or both. Nasopharyngeal-aspirate samples were obtained from all study patients to exclude common viruses including influenza viruses A and B, respiratory syncytial (RSV) virus, adenovirus, and parainfluenzavirus types 1, 2, and 3, using commercially-available immunofluorescence assays (IFA). Sputum and blood cultures were performed on all the cases to exclude bacterial or fungal infections. At 90 days, all the close contact relatives of the study SARS patients had their serum anti-coronavirus antibody measured by ELISA.

Nine non-smoking healthy volunteers (5 women and 4 men, aged 18 to 40 years) without evident current or past history of pulmonary diseases based on history as well as physical, chest radiographic and bronchoscopic examinations were selected as controls for this study. None of them had any upper respiratory tract infection within the last 6 weeks or was on antibiotics or other medications at the time of evaluation.

### Study protocol

The study protocol was approved by Chang Gung Memorial Hospital Ethical Committee. Informed consent was obtained from all the subjects. Treatment of SARS patients on admission to our unit included broad spectrum antibiotics to target common pathogens causing community-acquired pneumonia, according to current recommendations [13,14]. These patients received variable therapy regimens, including oral ribavirin (1 g twice a day for 5–7 days), or intravenous immunoglobulin (IVIG, 1 g/kg body weight/day for 2 days), pulse steroid therapy (methylprednisolone 500 mg twice a day for 3 days and then prednisolone 1 mg/kg body weight/day for 5 days), or maintenance corticosteroid therapy (prednisolone 10 mg twice a day for more than 3 weeks). Pulse steroid therapy was administered within 3 days of the onset of fever in some patients, depending on the attending physicians' decision irrespective of severity of presentation. Some patients who did not receive pulse steroid therapy were given a short course of corticosteroid therapy (hydrocortisone 100 mg 3 times/day for 3 days) if there was rapid deterioration of pulmonary infiltration or hypoxemia. Maintenance steroid therapy (prednisolone 10 mg per day for one week) was given after pulse or short course corticosteroid therapy in all patients. Two patients were intubated with ventilator support because of hypoxemic respiratory failure.

Patients underwent HRCT and BAL on the 60^th ^and 90^th ^day after the onset of disease. HRCT was performed with 1- to 2-mm collimation sections reconstructed by the use of a high spatial frequency algorithm using a (General Electric Medical Systems, Milwaukee, WI). The HRCT protocol consisted of thin sections obtained at 10-mm through the chest in a supine position without using intravenous contrast medium.

### Scoring of HRCT findings

The HRCT findings, as previously described [9], were categorized the predominant pattern as: normal attenuation; ground glass opacification (hazy areas of increased attenuation without obscuration of the underlying vessels); consolidation (homogeneous opacification of the parenchyma with obscuration of the underlying vessels); reticular pattern; mixed pattern (combination of consolidation, ground glass opacities and reticular opacities in the presence of architectural distortion); ground-glass attenuation with traction bronchiolectasis or bronchiectasis; and honeycomb pattern. The extent of involvement of each abnormality was assessed independently for each of three zones: upper (above the carina), middle (below carina up to the inferior pulmonary vein), and lower (below the inferior pulmonary vein). Each lung zone (total of 6 lung zones) was assigned a score, modified from previously described [9], based on the following: 0 when no involvement, 1 when <25% involvement, 2 when 25 <50% involvement; 3 when 50% <75% involvement and 4 when 75% involvement. Summation of scores provided overall lung involvement (maximal CT score 24). The grading of the patient's chest radiograph and HRCT was the consensus of two observers who were blind to clinical information of the patients.

### Fibreoptic bronchoscopy and BAL

BAL was performed on all the study subjects using six aliquots (50 ml each) of 0.9% saline solution as described previously [15]. Briefly, sterile saline solution was introduced into the subsegmental bronchus of the most severely involved lobe. The BAL fluid was retrieved and centrifuged. The supernatant was stored at -70°C until analysis and the cell pellet was washed and resuspended at 10^6 ^cells per ml. The cell viability and differential cell counts were determined. Total RNA and DNA were extracted from nasopharyngeal aspirates and cells retrieved by BAL with the Viral RNA minikit and QIAmp DNA minikit (QIAGEN, Hilden, Germany). Reverse-transcriptase (RT) PCR was done for influenza A, adenovirus, human metapneumovirus, and SARS-CoV as- described previously [16].

### Measurement of T cell subpopulations by flow cytometric analysis

BAL cells were simultaneously stained with fluorescein isothiocyanate or phycoerythrin-conjugated monoclonal antibodies (anti-IgG1, -IgG2a, -CD3, -CD4, -CD8, -CD19, -CD56) (Beckon Dickinson, Mountain View, CA) according to the manufacturer's protocol to identify the proportions of T lymphocytes, CD4, CD8 T cells, B cells and natural killer (NK) cells subpopulations respectively. The relative ratio of CD4 or CD8 in CD3-positive cells was assayed by a dual-color analysis. Data were acquired and analyzed using Becton Dickinson BD LYSYS II and Cytometric Bead Array (CBA) software (San Jose, CA).

### Levels of cytokine and chemokine in BAL fluid

The levels of cytokines and chemokines in BAL fluid were assayed using Becton Dickinson (BD) Cytometric Bead Array™ [17] (CBA; BD Biosciences, San Jose, CA) according to manufacturer's instructions with an antibody (PharMoingen, San Diego, CA) against one of five cytokines (Human Chemokine Kit I: CXCL8/IL-8, CCL5/RANTES, CXCL9/MIG, CCL2/MCP-1, CXCL10/IP-10, BD Biosciences,) or of the six cytokines (Human Inflammation Kit: IL-8, IL-1β, IL-6, IL-10, TNF-α, IL-12, BD Biosciences). Commercially available ELISA (R&D Systems, Minneapolis) was used for measurement of the growth factors, TGF-β, IGF-1 and EGF.

### Electron microscopic (EM) examination

We used a previously-described method for virus detection by electron microscopy [18]. Cells retrieved by BAL from SARS patients and normal subjects were centrifuged. The cell pellets were fixed, embedded and stained with 4% tannic acid and 0.5% uranyl acetate. The ultra-thin sections were cut from Epon-embedded blocks, stained with uranyl acetate and lead citrate, and examined using a transmission electron microscope (TEM) (H-500, Hitachi, Tokyo, Japan).

### Statistical analysis

Data are expressed as mean ± SEM. The baseline characteristics, disease and laboratory variables between groups were compared using the two-tailed Student *t*-test and chi-square test, respectively. Spearman rank test was used to determine correlations between HRCT scores and T cell numbers, and their subpopulations, as well as CD4/CD8 ratio. Univariate analyses to determine the factors responsible for persistence of HRCT abnormalities were primarily used for selection of variables, based on a *p *value <0.05. The significant variables were entered into a stepwise logistic regression analysis to determine the net effect for each predictor while controlling of the others. A *p*-value <0.05 was considered as statistically significant. Analysis was performed using SPSS software version 10.0 (Chicago, IL, USA).

## Results

### Study subjects

28 patients with confirmed diagnosis of SARS were admitted during the study period. Sixteen patients received intubation and ventilatory support for respiratory failure. Three died of intractable hypoxemic respiratory failure. Twenty-five patients recovered subsequently and were discharged from the hospital. No patient relapsed with either fever or new pulmonary infiltrates after discharge from the hospital. Twenty of the 25 patients were randomly selected into the protocol and twelve agreed to participate. These patients complied with the protocol at 60 days but at 90 days, only 10 of 12 patients agreed to have a repeat HRCT, and 6 of 12 patients had a follow-up bronchoscopy.

### Clinical manifestations

At 60 days, the commonest symptoms in SARS patients were general weakness (8 of 12 patients), exertional dyspnea (6 of 12 patients), joint pains (4 of 12 patients) and partial hair loss (11 of 12 patients). At 90 days, all the 12 SARS patients were well without any of the above-described symptoms. There was no detectable SARS-CoV antibody in the sera of close contact relatives of the study patients, even though SARS patients were not isolated after discharge from hospital from their close relatives.

### HRCT score

**Figure 1:**
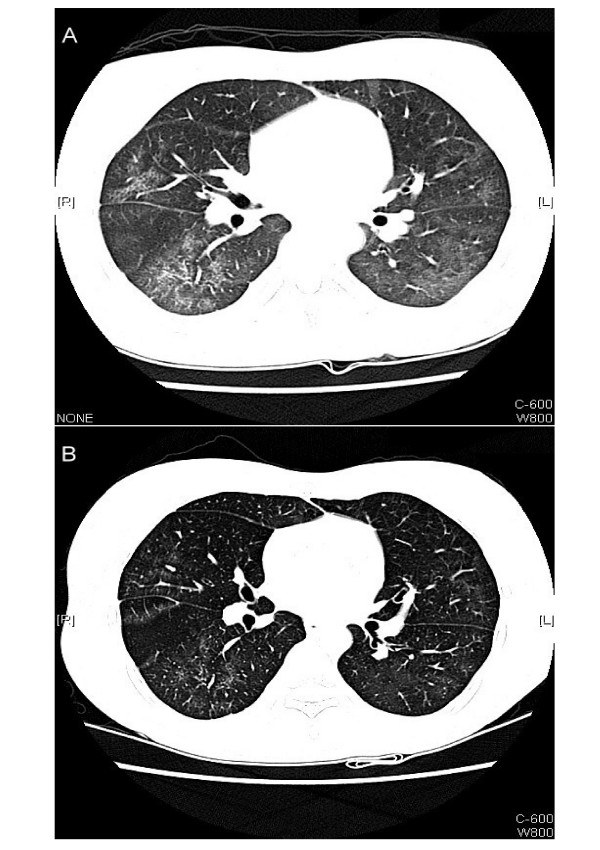
Residual abnormality on HRCT of a SARS patient with high HRCT score at 60 days (A). HRCT became almost normal at 90 days (B).

At 60 days, 5 SARS patients were found with an HRCT abnormality of <10 % of the total lung field. In 3, the score was zero, in one ground glass attenuation was found in 7.5% of total lung field, and in another, there was consolidation in 1.7% of total lung field. The other 7 SARS patients had HRCT abnormality > 10% of each lung field (a mean of 37.5 ± 7.9% involvement of total lung field) (Table 1). The most prominent HRCT findings in these patients were ground-glass attenuation (80.8 ± 12.2% of total abnormality on HRCT) and consolidation (13.6 ± 10.9% of total abnormality on HRCT). Honeycombing and bronchiectatic changes were found in only 3 SARS patients with high HRCT score (5.5 ± 2.7% of total abnormality on HRCT). Seven of 11 patients were found normal on their follow-up HRCT at 90 days (Table 2; Figure 1). Two of the patients had persistently high HRCT scores (Table 1). One with very high HRCT score at 60 days refused a follow-up HRCT.

**Table 1:** Individual HRCT score at 60 and 90 days, and electron microscopic findings in patients with SARS

	**HRCT score**		**Virus particle in AM by EM**	
		
	60 days	90 days	60 days	90 days
Case 1	0	0	-	N/D
Case 2	4	0	-	-
Case 3	0	0	-	N/D
Case 4	2	N/D	-	N/D
Case 5	3	0	-	N/D
Case 6	9	0	+	-
Case 7	12	3	+	N/D
Case 8	11	0	+	-
Case 9	12	7	+	-
Case 10	13	2	+	-
Case 11	15	12	+	N/D
Case 12	24	N/D	+	-
Mean ± SE	8.8 ± 2.1	2.4 ± 1.3*		

**Abbreviation: **HRCT, high resolution computed tomography; SARS, severe acute respiratory syndrome; AM, alveolar macrophage; EM, electron microscopy; N/D, not done.

*p *< 0.01 indicates a comparison of HRCT score between 60 days and 90 days in corresponding group.

**Table 2:** Univariate and multivariate analysis: predictors based on presence of virus particle and lung involvement in patients with SARS.

**Factor**	**Low HRCT score and Absence of virus particle** **(n = 5)** **no. (%)**	**High HRCT score and Presence of virus particle** **(n = 7)** **no. (%)**	**Univariate analysis**			**Multivariate analysis**
			
			***P *value**	**Odd ratio**	**95% confidence interval**	***P *value**
Age, year	25.6 ± 4.2	34.9 ± 2.9	0.09	-	-	-
Female gender	5 (100%)	4 (57.1%)	0.09	1.75	0.92–3.32	-
Titer of Anti-CoV IgG (OD) *	0.8 ± 0.2	1.3 ± 0.1	0.04	-	-	1.0
Days of fever	4.2 ± 0.5	11.0 ± 1.0	0.0003	-	-	0.011
Positive PCR	2 (28.6%)	5 (71.4%)	0.276	3.75	0.33–42.47	-
Use of ribavirin	4 (57.1%)	6 (85.7%)	0.79	1.50	0.71–31.58	-
Use of IVIG	4 (57.1%)	6 (85.7%)	0.79	1.50	0.71–31.58	-
Pulse corticosteroid therapy	0 (0%)	4 (57.1%)	0.04	2.33	0.99–5.49	0.004
Maintenance corticosteroid therapy	0 (0%)	3 (42.9%)	0.09	1.75	0.92–3.32	-
Need for intubation	1 (14.3%)	1 (14.3%)	0.79	0.67	0.03–14.03	-

**Abbreviation: **HRCT, high resolution computed tomography; SARS, severe acute respiratory syndrome; IVIG, intravenous immunoglobulin; CoV, coronavirus; OD, optical density; PCR, polymerase chain reaction. Data are shown as mean ± SEM.

*The cut value of positive SARS infection is 0.12 OD.

### Factors associated with residual HRCT abnormalities at day 60

The residual abnormality on HRCT at 60 days was related to the clinical course. Univariate analysis identified 3 factors associated with the residual abnormality on HRCT. There were a greater proportion of patients receiving pulse steroid therapy (4 of 7) in patients with high HRCT score (Table 2). In contrast, none of the patients with low HRCT score received pulse steroid therapy (Table 2). There was no significant difference in other therapy, including maintenance or short course corticosteroid therapy, IVIG or ribavirin, between patients with high HRCT score and those with low HRCT score (Table 2). Patients with high HRCT score had significantly longer course of fever and higher serum SARS-CoV antibody titer when compared to those in patients with low HRCT score (Table 2).

### Inflammatory profiles of BAL fluid

**Figure 2:**
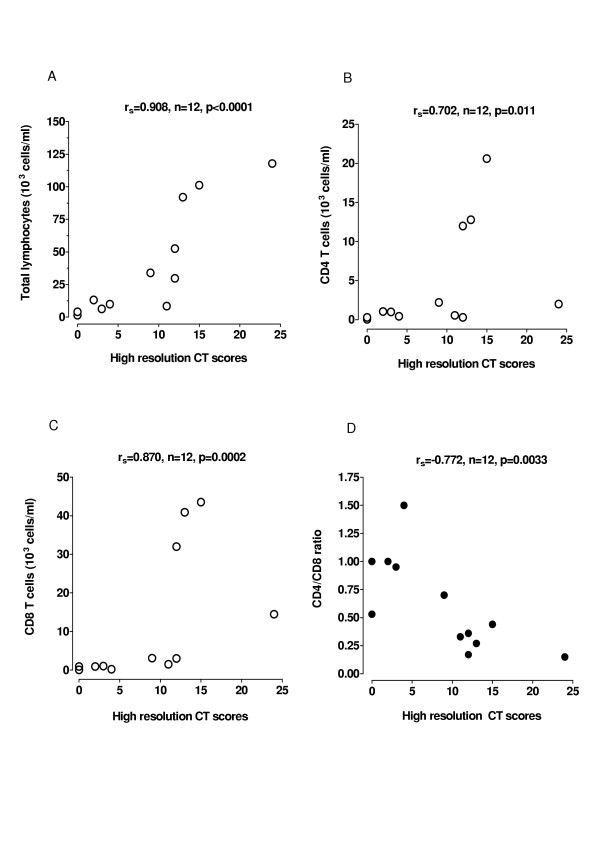
Correlation of the cell counts of (A) total lymphocytes, (B) CD4 and (C) CD8 T cells, or the (D) CD4/CD8 ratio with HRCT scores in SARS patients. The analysis is made by Spearman rank test and the number and significance are indicated.

At 60 days, compared to normal subjects, there was a significant increase in total cell counts in BAL fluid from SARS patients (Table 3) with a significant increase in alveolar macrophages (AM) and lymphocytes., The proportion of CD8+ T cells was increased to a greater extent than CD4+ T cells, leading to a significant decrease in CD4/CD8 ratio (Table 4). There was also a significant increase in the proportion of NK cells in SARS patients (Table 4). There was no significant difference in B lymphocytes between SARS patients with low or high HRCT scores and normal subjects. HRCT scores were highly correlated with the cell counts of total lymphocyte, CD4+ and CD8+ T cells, and inversely related to the CD4/CD8 ratio (Figure 2). At 90 days, the cellular profiles in BAL fluid of 6 SARS patients were significantly improved compared with those at 60 days, with near normalization (Tables 3, 4).

**Table 3:** Characteristics of bronchoalveolar lavage in normal subjects and patients with SARS

	**Normal subjects**	**SARS patients**	
	
		**60 days**	**90 days**
	**(n = 9)**	**(n = 12)**	**(n = 6)**
Age (years)	24.1 ± 2.2	34.0 ± 2.7*	36.6 ± 3.9
Female gender	5	4	3
Cellularity (10^4 ^cells/ml)	9.6 ± 0.9	32.9 ± 9.0*	26.2 ± 9.1
Cell viability (%)	91.5 ± 4.3	90.4 ± 1.3	91.6 ± 1.8
AM (%)	93.2 ± 1.2	88.8 ± 1.2*	95.0 ± 0.6†
AM (10^4 ^cells/ml)	8.9 ± 0.8	29.0 ± 7.8*	25.1 ± 9.8
Lymphocytes (%)	5.9 ± 1.2	10.2 ± 1.2*	4.1 ± 0.5†
Lymphocytes (10^4 ^cells/ml)	0.6 ± 0.1	3.8 ± 1.2*	1.0 ± 0.2†
Neutrophils (%)	0.9 ± 0.2	0.7 ± 0.2	0.9 ± 0.6
Neutrophils (10^4 ^cells/ml)	0.1 ± 0.02	0.2 ± 0.1	0.2 ± 0.1
Eosinophils (%)	0.1 ± 0.1	0.3 ± 0.2	0.0 ± 0.0
Eosinophils (10^4 ^cells/ml)	0.01 ± 0.01	0.05 ± 0.04	0.0 ± 0.0

**Abbreviation: **AM, alveolar macrophages; HRCT, high resolution computed tomography; SARS, severe acute respiratory syndrome.

*p < 0.01 compared with normal subjects.

† *p *< 0.05 compared with SARS patients at 60 days.

Data are mean ± SEM.

**Table 4:** Lymphocyte subpopulations in bronchoalveolar lavage from normal subjects and patients with SARS

	**Normal subjects**	**SARS patients**	
		
		**60 days**	**90 days**
	**(n = 9)**	**(n = 12)**	**(n = 6)**
Lymphocytes (10^3 ^cells/ml)	5.8 ± 1.4	39.2 ± 12.1*	9.7 ± 2.4^†^
CD3 cells (%)	39.7 ± 6.4	33.1 ± 6.7	37.8 ± 6.1
CD3 cells (10^3 ^cells/ml)	2.4 ± 0.5	16.3 ± 6.4*	3.3 ± 0.7
CD4 cells (%)	9.2 ± 2.6	8.7 ± 2.2	10.4 ± 4.3
CD4 cells (10^3 ^cells/ml)	1.2 ± 0.3	4.4 ± 2.0*	0.8 ± 0.3
CD8 cells (%)	6.6 ± 2.6	20.1 ± 5.5*	13.2 ± 3.3
CD8 cells (10^3 ^cells/ml)	0.7 ± 0.1	11.8 ± 4.7*	1.1 ± 0.2^†^
CD4/CD8 (ratio)	1.89 ± 0.22	0.62 ± 0.12*	0.73 ± 0.12^†^
B cells (%)	6.7 ± 1.2	3.2 ± 0.8	2.8 ± 0.6
B cells (10^3 ^cells/ml)	0.4 ± 0.1	1.4 ± 0.7	0.3 ± 0.1
NK cells (%)	1.8 ± 0.2	8.8 ± 2.6*	5.8 ± 2.1
NK cells (10^3 ^cells/ml)	0.1 ± 0.03	4.0 ± 2.4**	0.3 ± 0.1^†^

**Abbreviation: **HRCT, high resolution computed tomography; NK, natural killer.

*p < 0.05, ** *p *< 0.01 compared with normal subjects.

†*p *< 0.05 compared with SARS patients at 60 days.

Data are mean ± SEM.

### Cytokine and chemokine level in BAL fluid

At 60 days, SARS patients had a significantly higher level of chemokines, IL-8, MCP-1, and RANTES (Table 5), and of pro-inflammatory cytokines, TNF-α and IL-6. However, the growth factors, transforming growth factor-β (TGF-β), epidermal growth factor (EGF), insulin-like growth factor-1 (IGF-1), were not increased (Table 5).

**Table 5:** Cytokine and chemokine levels in bronchoalveolar lavage from normal subjects and SARS patients

	**Normal subjects (n = 9)**	**SARS patients (n = 12)**
CXCL10/IP-10 (pg/ml)	95.8 ± 25.7	133.1 ± 37.5
CXCL9/MIG (pg/ml)	20.2 ± 6.5	53.1 ± 14.1*
IL-8 (pg/ml)	1.5 ± 0.2	6.3 ± 1.0**
CCL2/MCP-1 (pg/ml)	2.4 ± 0.8	9.0 ± 1.2**
CCL5/RANTES (pg/ml)	1.0 ± 0.4	34.6 ± 9.3**
TNF-α (pg/ml)	0.004 ± 0.002	1.1 ± 0.3*
IL-1β (pg/ml)	0.00 ± 0.00	2.5 ± 1.8
IL-6 (pg/ml)	0.001 ± 0.001	1.7 ± 0.5**
IFN-γ (pg/ml)	0.0 ± 0.0	0.4 ± 0.3
IL-2 (pg/ml)	0.00 ± 0.00	0.4 ± 0.2
TGF-β (pg/ml)	9.6 ± 2.9	15.4 ± 4.6
IGF-1 (ng/ml)	0.06 ± 0.03	0.07 ± 0.05
EGF (pg/ml)	0.0 ± 0.0	0.0 ± 0.0

* *p *< 0.05, ** *p *< 0.01 compared with normal subjects.

Data are shown as mean ± SEM.

### Virus detection

The 12 enrolled patients had serological evidence of recent infection with the SARS-CoV and in seven, viral RNA was detected in samples taken from nasopharyngeal aspirate or stool. However, viral RNA was not detectable in the stool or nasopharyngeal aspirate of any of the SARS patients at 60 days. Healthy controls had no evidence of SARS-CoV antibody or RNA in the serum or the respiratory tract. There were no detectable common viruses including influenza viruses A and B, RSV virus, adenovirus, human metapneumovirus, and parainfluenzavirus types 1, 2, and 3, using IFA for nasopharyngeal aspirates or using RT-PCR assay for cells retrieved by BAL at 60 or 90 days. Serological studies for *Clamydia, Mycoplasma *or *Legionella *were negative in all subjects.

**Figure 3:**
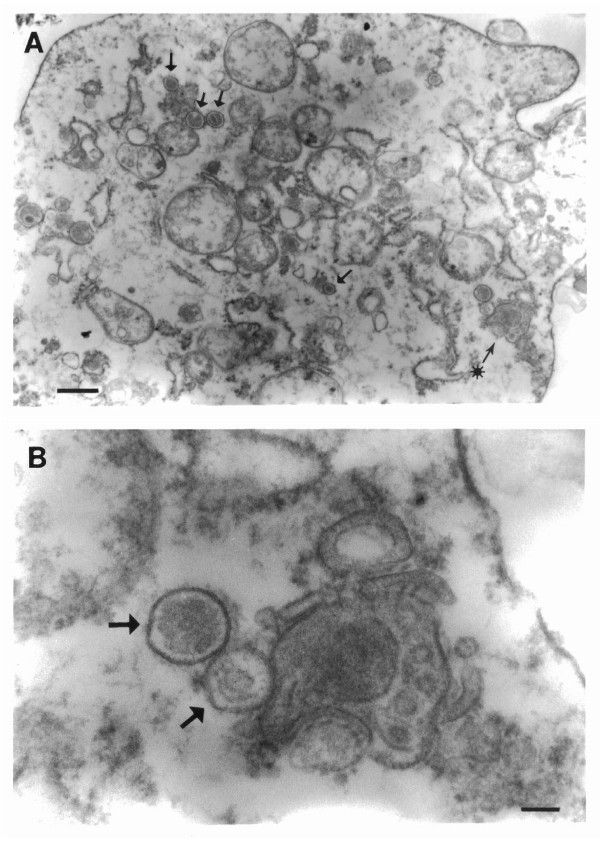
Ultrastructural characteristics of a Coronavirus-Infected cell in BAL fluid from a SARS patient at 60 days, with several intracellular particles. The virions are indicated by the arrowheads in Panel A. Panel B shows the area indicated by the asterisk in Panel A at higher magnification. The bar in Panel A (500 nm) and Panel B (100 nm) is indicated.

At 60 days, EM examination of BAL fluid revealed many coronavirus-infected alveolar macrophages with intracellular viral particles in 7 of 12 patients (Figure 3; Table 1). These patients had the high HRCT scan scores. Coronavirus infected cells were not detected in any of SARS patients with low HRCT score or in normal subjects (Table 1). RT-PCR amplification of coronavirus nucleic acids was positive in 3 of 7 patients with high HRCT score, but in none of patients with low HRCT score or normal subjects. At 90 days, EM examination did not detect any coronavirus-infected cells in 6 SARS patients, in 5 of the 6, viral inclusions were found in AM at day 60 (Table 1). One patient with persistent high HRCT score (case 12) refused follow-up BAL study at 90 days.

## Discussion

This study was performed during the last epidemic of SARS in Taiwan, and the number of patients recruited has been limited. The epidemic did not recur during 2004, and there have been no further cases of SARS in Taiwan, such that we were not able to increase the number of patients in this study. Despite the relatively low numbers, our observations indicate that there are persistent important inflammatory and radiological abnormalities in some patients who have recovered from acute SARS at 60 days after the illness. These changes were those of ground-glass or/and consolidation abnormalities which may be overlooked on examination of plain chest radiographs. The BAL fluid examination performed for the first time in recovering SARS patients confirmed the presence of an on-going active inflammatory process in most patients with increased macrophages, NK cells and T cells, and augmented levels of chemokines and pro-inflammatory cytokines. These inflammatory responses may be elicited by the persistent presence of coronavirus in alveolar macrophages, since the patients with the highest HRCT changes had coronaviruses present and there was no evidence of bacterial or other viral infection in these patients.

Most viral diseases are characterized by the development of a specific infiltration consisting predominantly of mononuclear leukocytes while neutrophils are absent [19]. The most striking features of alveolar inflammation in patients were increased numbers of alveolar macrophages, T lymphocytes and NK cells, with a striking decrease in CD4/CD8 ratio. The HRCT score was highly correlated with T lymphocyte numbers and their subpopulations, and was inversely related to CD4/CD8 ratio. CD8+ T cells may act as cytotoxic cells and are key effectors of virus clearance [20]. The concurrent increase in CD4+ T cells may promote the clonal expansion of virus-specific CD8+ T cells and is essential for maintaining continued CD8+ T cells surveillance and effector capacity [19].

Exposure of monocytes or macrophages to viruses causes the release of proinflammatory cytokines, such as TNF-α, IL-1, and IL-6, and chemokines [20-22] as well as members of the CC-chemokine subfamily such as MIP-lα, MCP-1, and RANTES which preferentially attract monocytes and lymphocytes [22]. The CXC-chemokines, such as IL-8 or GRO-α, are major neutrophil chemoattractants [23]. MIG/CXC chemokine ligand (CXCL) 9 and IP-10/CXCL10, both inducible by interferon-γ, are ELR-negative CXC chemokines and are potent chemoattractants for mononuclear cells, specifically activated T lymphocytes and NK cells [24]. In this report, we demonstrated elevated levels of TNF-α, IL-6, MCP-1, RANTES and IL-8 in BAL fluid in SARS patients compared to those of normal subjects. Increased secretion of TNF-α and IL-6 may be derived from virus-infected macrophages or from CD4+ or CD8+ T cells, and these cytokines may promote T-lymphocyte extravasation and macrophage activation [19], but such processes may not be sufficient on their own to recruit and activate mononuclear cells in virus-infected lungs. The increased levels of MCP-1 and RANTES in BAL fluid of all SARS patients may be responsible for the generation of mononuclear infiltrates observed after coronavirus infection. IP-10 and MIG, whose levels are also increase in SARS patients, recruit monocytes and macrophages, NK cells and activated, but not resting T lymphocytes [25,26].

Although there were increased levels of IL-8 in BAL fluid in SARS patients, the number of neutrophils in BALF were sparse. The absence of neutrophil infiltration in influenza A virus or respiratory syncytial viruses (RSV) infection is attributed to the suppression of neutrophil attracting CXC-chemokines or by induction of IL-10 [27]. However, IL-8 production can be induced by measles virus infection of fibroblasts [28] and by influenza A virus, RSV and rhinovirus in pulmonary epithelial cells or AM [28-31]. The reasons for the lack of neutrophil recruitment in response to elevated IL-8 levels in SARS patients are not known and this deserves further investigation.

Despite the presence of virus in AM at 60 days when patients had already been discharged from hospital, these patients were not infectious, because none of their close contact relatives developed any detectable SARS-CoV antibody in their sera. The HRCT and the clinical course until the 90th day of illness did not suggest any evidence of pulmonary fibrosis in SARS patients. This was in accord with the low level of cytokines and growth factors responsible for tissue repairing and fibrosis [32], such as IL-1β, TGF-β, IGF-1, and EGF detected in BAL fluid. However, evidence of fibrosis on HRCT has been obtained on HRCT scans particularly in patients with very severe disease during the acute phase of SARS [33].

The use of corticosteroids together with ribavirin has been reported to confer clinical benefit, although randomized clinical trials to support its clinical efficacy are not available. Pulse steroid therapy was reported to lead to less oxygen requirement, better radiographic outcome, and less likelihood to require rescue pulse steroid therapy than their counterparts [34]. However, corticosteroids have been shown to increase mortality of pneumovirus-infected mice by accelerating replication of virus [35]. Pulse steroid therapy was also reported to be associated with residual abnormality on HRCT in SARS patients after discharge from hospital [10]. In the current retrospective, non-randomized series, pulse steroid therapy appeared to be associated with delayed resolution of pneumonitis. We have planned a prospective future study on investigating the effect of pulse steroid therapy in case of future outbreaks of SARS. This is because this issue is extremely important in outcome from SARS.

In conclusion, a proportion of recovered SARS patients have delayed resolution of pneumonitis and delayed clearance of coronavirus in the alveolar space at day 60. This was associated with persistent inflammatory response characterized by macrophages, T cells particularly CD8+ T cells, and NK cells, and by increase in cytokines and chemokines. This host inflammatory response against SARS-CoV infection may contribute to persistent HRCT abnormalities during recovery phase of SARS. On the other hand, we found no evidence of pulmonary fibrosis in SARS patients during recovery. At day 90, many of the abnormalities have disappeared. Patients recovering from SARS need to be followed up for at least 3 months after the infection.
